# Sex Differences in Cardiometabolic Risk in Adults With Type 2 Diabetes Mellitus Attending a Regional Referral Hospital in Kenya: A Cross-Sectional Study

**DOI:** 10.7759/cureus.103445

**Published:** 2026-02-11

**Authors:** Elizabeth W Maina, Samuel T Kimani, James Mwaura

**Affiliations:** 1 Nursing, University of Nairobi, Nairobi, KEN; 2 Nursing, University of Embu, Embu, KEN

**Keywords:** adults, cardiometabolic risks, kenya, sex differences, type 2 diabetes mellitus

## Abstract

Introduction: Type 2 diabetes mellitus (T2DM) is associated with an increased risk of cardiometabolic complications, with attendant morbidity and mortality worldwide. Evidence suggests sex-linked disparities in cardiometabolic risks due to biological, social, behavioral, and socioeconomic determinants. However, sex-linked cardiometabolic risks among adults with T2DM in Kenya are poorly understood. This study examined sex differences in cardiometabolic risk factors among adults with T2DM attending Embu County Referral Hospital in Kenya.

Materials and methods: This cross-sectional study included 136 adults with T2DM aged ≥18 years who were diagnosed at least six months earlier. Data were collected using a researcher-administered questionnaire. Physiological and anthropometric measurements, including body mass index (BMI), waist-hip ratio (WHR), blood pressure (BP), random blood glucose (RBG), and glycated haemoglobin (HbA1c), were measured. Data analysis involved descriptive and inferential statistics, including age-adjusted logistic regression analyses of cardiometabolic risks by sex. The p-value was set at ≤0.05.

Results: Participants were predominantly female (69.9%), with a mean (±SD) age of 56.34 (±13.83) years. Females were more likely than males to have a prior hypertension diagnosis (73.33% vs. 26.67%; p < 0.001), be obese (88.9% vs. 11.1%; p = 0.003), have a higher waist circumference (93.6% vs. 6.4%; p < 0.001), and have a higher WHR (97.7% vs. 2.3%; p < 0.001). No significant sex differences were observed in RBG, HbA1c, or BP measurements. After age adjustment, sex was not independently associated with the cardiometabolic risks. Adults aged 50-65 years had significantly lower odds of high BMI compared with those aged <50 years (AOR = 0.35, 95% CI: 0.13-0.92; p = 0.033). Females showed a lower, but statistically nonsignificant, adjusted odds of poor glycemic control.

Conclusions: Notable sex differences in key sociodemographic characteristics and cardiometabolic markers were observed, though attenuated by age adjustment. These findings underscore the need for age- and sex-specific preventive and management strategies to improve cardiometabolic outcomes.

## Introduction

Diabetes mellitus remains a global health concern, with recent estimates indicating that approximately 589 million adults aged 20-79 years are living with diabetes worldwide. Regional projections suggest a disproportionate rise in low- and middle-income regions, particularly in Africa [1]. Type 2 diabetes mellitus (T2DM), which accounts for the majority of diabetes cases, is closely associated with cardiometabolic risk factors, including hypertension, obesity, dyslipidemia, and insulin resistance. These factors substantially increase the risk of cardiovascular disease (CVD), with a resultant increase in morbidity and mortality among people diagnosed with T2DM [2,3].

Growing evidence shows that cardiometabolic risks and their clinical manifestations differ significantly between males and females with T2DM. Studies from high-income settings have consistently reported sex-based disparities in the prevalence, progression, and outcomes of cardiometabolic complications [4,5]. While men generally develop T2DM at a younger age and at a lower body mass index (BMI), women with T2DM tend to experience a greater relative increase in cardiovascular risk compared with women without diabetes [6,7]. This difference may be partly explained by biological factors, such as the cardioprotective effects of estrogen, which are lost after menopause [4,7]. Additionally, males and females differ in body fat distribution, with women more likely to accumulate subcutaneous fat and men more likely to accumulate visceral fat. The accumulation of abdominal and visceral fat has been strongly associated with increased cardiometabolic risk and adverse cardiovascular outcomes [6].

Furthermore, psychosocial, economic, and cultural factors play a significant role in shaping sex differences in cardiometabolic risk profiles. Social determinants of health, such as health-seeking behavior, access to healthcare services, and economic and cultural expectations, differ between sexes and influence the management of T2DM and the development of associated complications [8,9]. For example, women may face barriers related to caregiving roles and economic constraints. At the same time, men often demonstrate poorer health-seeking behavior and lower adherence to long-term care compared to women [10,11]. The interaction between biological factors and gendered determinants of health further widens sex disparities in cardiometabolic risks and outcomes, underscoring the need for a comprehensive understanding that integrates both dimensions [8].

Despite extensive evidence from high-income countries, sex-specific cardiometabolic risks in T2DM remain underexplored in sub-Saharan Africa, resulting in limited data. This is particularly concerning given the region’s unique challenges, including constrained healthcare resources, limited access to early diagnosis and specialized care, and a high burden of T2DM and undiagnosed cardiometabolic conditions, which may modify sex-specific risk patterns. In Kenya, diabetes clinics have been established as essential points of care for individuals with T2DM. However, routine clinical data and research evidence rarely disaggregate cardiometabolic risk profiles by sex, limiting the development of targeted, sex-specific prevention and management strategies. Understanding sex differences in cardiometabolic risks in this context, which may be generalizable to similar settings, is therefore critical, as it can inform clinical practice, guide policy, and improve long-term outcomes for both men and women living with T2DM.

This study, therefore, aimed to examine sex differences in cardiometabolic risks among adults with T2DM attending Embu County Referral Hospital in Kenya.

## Materials and methods

Study design

The study employed a cross-sectional design to collect quantitative data on the variables of interest, including BMI, waist-hip ratio (WHR), blood pressure (BP), random blood glucose (RBG), and glycated hemoglobin (HbA1c). Data were collected at a single point in time; therefore, causal relationships between variables were not established.

Study settings and sample

The study was conducted in the diabetes clinic at Embu County Referral Hospital, a regional hospital in Kenya. The clinic serves residents of Embu County and neighboring counties, including Kirinyaga, Kitui, Tharaka Nithi, and Machakos. Owing to its proximity to Embu town, the clinic primarily serves urban and suburban populations.

The study involved male and female adults with T2DM. Participant recruitment and data collection were carried out between July and August 2022. Recruitment occurred over four weeks during routine clinic visits, drawing from the clinic’s database of 200 registered patients on follow-up. We recruited all eligible participants attending the clinic during the study period (n = 136). As this was a clinic-based cross-sectional study using convenience sampling, no a priori sample size calculation was performed.

Inclusion and exclusion criteria

The inclusion criteria were adults aged 18 years or older with primary T2DM who were attending clinic follow-up and provided informed consent. Pregnant and lactating women were excluded due to the possibility of gestational diabetes.

Data collection tools and procedures

A researcher-assisted structured questionnaire was used to collect data on sociodemographic characteristics, history of physician-diagnosed hypertension, HbA1c levels, BP, and anthropometric measurements of the participants.

Validity and reliability of the study tools

The study tool was developed by the research team and validated by an expert in diabetes research. The feedback provided was incorporated into the final tool. To assess reliability, Cohen’s kappa statistic was used, with a test-retest coefficient of 0.8 considered acceptable. The instruments were also pretested with 10 participants from another diabetes clinic in the county that offered similar services, and minor modifications were made to enhance clarity.

To ensure the reliability and validity of study procedures, measurements were taken by the same team of research assistants and laboratory technicians using standard techniques and the same calibrated equipment. Before participant recruitment and data collection, the research team provided training to research assistants and laboratory technicians on ethical research conduct and study procedures.

RBG, HbA1c, BP, and anthropometric measurements

Measurements were taken using standard techniques described in a previous study [2]. The assessments included RBG, systolic and diastolic BP, HbA1c, body weight, height, waist circumference (WC), and hip circumference. WHR and BMI were calculated from the relevant measurements.

Variable definitions and outcome measures

Good glycemic control was defined as HbA1c < 7%, while HbA1c ≥ 7% indicated poor control [12]. RBG values ≥ 11.1 mmol/L were classified as high/uncontrolled [12]. The presence of elevated HbA1c and/or uncontrolled RBG denoted poor glycemic control.

BMI categories included underweight (<18.5), normal (18.5-24.9), overweight (≥25), and obese (≥30) [13], with overweight/obesity regarded as high risk for cardiovascular complications [14]. Central obesity was defined as a high WC (>94 cm in men and >80 cm in women) [13,15]. WHR values <0.8 for women and <0.9-0.95 for men indicated low risk, 0.8-0.85 for women and 0.9-1.0 for men indicated moderate risk, while >0.90 for men and >0.85 for women indicated a high cardiometabolic risk [15].

High/uncontrolled BP was defined as systolic BP ≥140 mmHg or diastolic BP ≥90 mmHg on BP measurement [16]. However, in the estimation of the prevalence of hypertension at the time of recruitment, participants with BP below these limits but who reported a previous hypertension diagnosis from a physician or were on antihypertensive medication were recorded as having hypertension.

Statistical analysis

Descriptive statistics (means, standard deviations, frequencies, and percentages) were used to summarize the sociodemographic characteristics and cardiometabolic risks of the sample using SPSS version 24.0 (IBM Corp. Released 2016. IBM SPSS Statistics for Windows, Version 24.0. Armonk, NY: IBM Corp.). Relationships between sex and sociodemographic characteristics, and between sex and cardiometabolic risks, were tested using the chi-square test of independence and Fisher’s exact test when a cell had an expected value of five or less. Age-adjusted binary logistic regression models were fitted for each outcome to examine the independent association between sex and cardiometabolic risk factors. Adjusted odds ratios (AORs) with 95% confidence intervals (CIs) were reported. The reference categories were male for sex and <50 years for age. A p-value ≤0.05 was considered significant.

Ethics approval

Ethics approval was obtained from the Kenyatta National Hospital, University of Nairobi Ethics and Research Committee (approval number: P140/03/2021). Permission to collect data was obtained from the administration of Embu County Referral Hospital. Written informed consent was obtained from all the participants prior to data collection. The study was conducted in accordance with the ethical principles of the Declaration of Helsinki and adhered to the STROBE guidelines for observational research. Participants' anonymity, privacy, and confidentiality were ensured throughout data collection and after. All data was securely stored in lockable cabinets and on password-protected computers, accessible only to authorized members of the research team.

## Results

Sex differences in sociodemographic characteristics of the participants

Sex differences in participants' sociodemographic characteristics are presented in Table 1. A total of 136 participants were recruited. The mean (±SD) age was 56.34 (±13.83) years; most participants were female (69.9%), married (78.7%), and Protestant (74.3%). A majority (45.6%) were self-employed, and 47.1% had attained primary-level education or no formal education. Further analysis using chi-square and Fisher’s exact tests revealed significant relationships between sex and age (p = 0.001), marital status (p = 0.021), and employment status (p = 0.021). Males were more likely to be older (>65), married, and employed. However, there were no significant differences in religion or educational attainment by sex (p > 0.05).

**Table 1:** Sex differences in sociodemographic characteristics of the participants * Analyses using Fisher's exact test SD: standard deviation

Characteristic	Total (n = 136)	Male (n = 41, 30.1%)	Female (n = 95, 69.9%)	χ^2^ test	Df	p-value
Mean age (±SD)	56.34 (13.83)					
Age group in years (n,%)						
<50	47 (34.6)	12 (29.3)	35 (37.35)	14.42	2	0.001
50-65	41 (30.1)	6 (14.6)	35 (37.35)			
>65	48 (35.3)	23 (56.1)	25 (25.30)			
Marital status (n,%)						
Married	107 (78.7)	39 (95.14)	68 (71.6)	11.57	4	0.021*
Single	12 (8.8)	1 (2.43)	11 (11.6)			
Divorced/separated/widowed	17 (12.5)	1 (2.43)	16 (16.8)			
Religion (n,%)						
Roman catholic	35 (25.7)	11 (26.8)	24 (25.3)	0.037	1	0.848
Protestant	101 (74.3)	30 (73.2)	71 (74.7)			
Education (n,%)						
No formal education to the primary level	64 (47.1)	14 (34.1)	50 (52.6)	8.53	4	0.074
Secondary level	49 (36.0)	15 (36.6)	34 (35.8)			
Tertiary level	23 (16.9)	12 (29.3)	11 (11.6)			
Employment status (n,%)						
Formally employed	11 (8.1)	4 (9.8)	7 (7.37)	11.58	4	0.021*
Casually employed	7 (5.1)	2 (4.9)	5 (5.27)			
Self-employed	62 (45.6)	15 (36.6)	47 (49.47)			
Unemployed	56 (41.2)	20 (48.8)	36 (37.89)			

Sex differences in the history of physician-diagnosed hypertension

**Figure 1:**
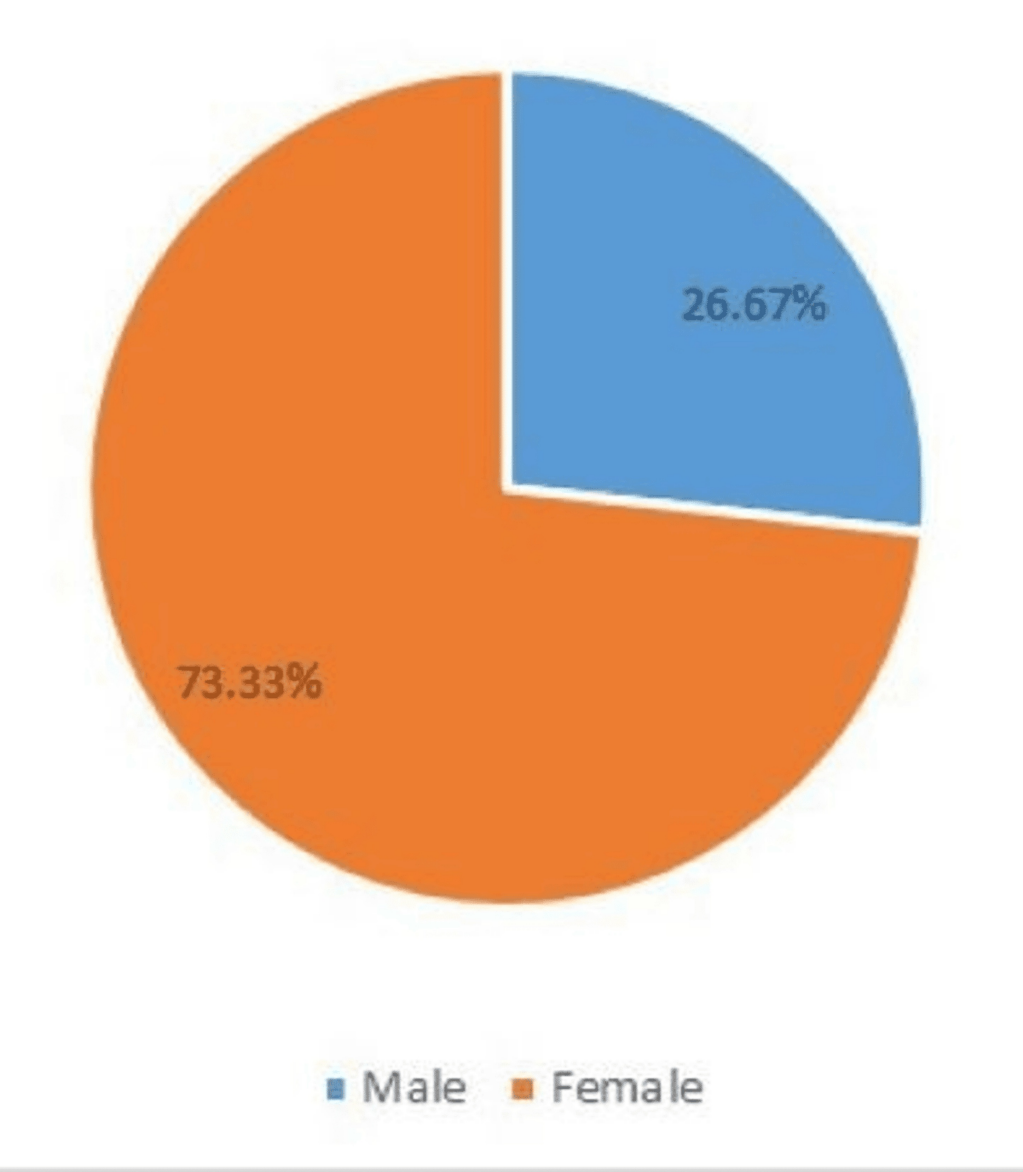
History of physician-diagnosed hypertension across sex

The history of physician-diagnosed hypertension in male and female participants across sexes is presented in Figure 1. The majority of the total participants (66.18%, n = 90) had physician-diagnosed hypertension at the time of recruitment. Analysis using the chi-square (χ2) test of independence revealed that significantly more females (73.33%, n = 66) than males (26.67%, n = 24) were hypertensive at the time of recruitment (p < 0.001).

Sex differences in cardiometabolic risk factors

Sex differences in cardiometabolic risk factors are shown in Table 2. Analysis using the chi-square (χ2) test of independence revealed that female participants were significantly more at risk of CVD. Female participants were significantly more likely to be obese (88.9% vs. 11.1%; p = 0.003) compared with male participants. Similarly, a significantly higher proportion of females had elevated WC (93.6% vs. 6.4%; p < 0.001). As estimated by WHR classification, cardiometabolic risk was also significantly higher among females (97.7% vs. 2.3%; p < 0.001). Although levels of RBG (75.5% vs. 24.5%), HbA1c (69.8% vs. 30.2%), and BP (72.2% vs. 27.9%) were higher among females than males, these differences were not statistically significant (p > 0.05).

**Table 2:** Sex differences in cardiometabolic risk factors (n, %) * Analyses using Fisher's exact test BMI: body mass index, WC: waist circumference, WHR: waist-hip ratio, RBG: random blood glucose, HbA1c: glycated hemoglobin, BP: blood pressure

Measurements	Sex		Total (n, %)	χ^2^ test	Df	p-value
	Male (n, %)	Female (n, %)				
BMI (kg/m^2^)						
Normal	18 (39.1)	28 (60.9)	46 (100.0)	11.581	2	0.003
Overweight	18 (40.0)	27 (60.0)	45 (100.0)			
Obese	5 (11.1)	40 (88.9)	45 (100.0)			
Total	41 (30.1)	95 (69.9)	136 (100.0)			
WC (cm)						
Normal	36 (62.1)	22 (37.9)	58 (100.0)	48.935	1	<0.001
High	5 (6.4)	73 (93.6)	78 (100.0)			
Total	41 (30.1)	95 (69.9)	136 (100.0)			
WHR						
Low risk	17 (89.5)	2 (10.5)	19 (100.0)	96.934	1	<0.001*
Moderate risk	22 (73.3)	8 (26.7)	30 (100.0)			
High risk	2 (2.3)	85 (97.7)	87 (100.0)			
Total	41 (30.1)	95 (69.9)	136 (100.0)			
RBG level (mmol/L)						
Normal	28 (33.7)	55 (66.3)	83 (100.0)	1.303	1	0.254
High	13 (24.5)	40 (75.5)	53 (100.0)			
Total	41 (30.1)	95 (69.9)	136 (100.0)			
HbA1c (%)						
Normal	15 (30.0)	35 (70.0)	50 (100.0)	0.001	1	0.977
High	26 (30.2)	60 (69.8)	86 (100.0)			
Total	41 (30.1)	95 (69.9)	136 (100.0)			
BP (mmHg)						
Normal	22 (32.4)	46 (67.6)	68 (100.0)	0.314	1	0.575
High	19 (27.9)	49 (72.2)	68 (100.0)			
Total	41 (30.1)	95 (69.9)	136 (100.0)			

Age-adjusted logistic regression analysis of sex differences in cardiometabolic risk factors

The independent association of sex with cardiometabolic risk factors was examined using age-adjusted logistic regression (Table 3). While unadjusted analyses suggested higher adiposity among females, these associations were attenuated after adjusting for age, indicating that age partially confounds the association between sex and cardiometabolic risk factors.

After adjusting for age categories (<50, 50-65, ≥65 years), sex was not independently associated with any cardiometabolic outcome. Females had lower odds of high BMI compared with males (AOR = 0.65; 95% CI: 0.29-1.49; p = 0.313). Age 50-65, however, was associated with significantly lower odds of high BMI relative to <50 years (AOR = 0.35; 95% CI: 0.13-0.92; p = 0.033). Additionally, females had lower, but non-significant, odds of both high WC (AOR = 0.42; 95% CI: 0.15-1.17; p = 0.097) and high-risk WHR (AOR = 0.27; 95% CI: 0.05-1.58; p = 0.147). There was, however, no significant association with age for either WC or WHR. Furthermore, no significant association was observed for sex or age for both RBG (AOR = 0.70; 95% CI: 0.31-1.57; p = 0.389) and HbA1c (AOR = 1.58; 95% CI: 0.69-3.65; p = 0.282). On the other hand, females had a higher but non-significant odds of high BP (AOR = 1.66; 95% CI: 0.75-3.65; p = 0.211), with no significant association for age.

**Table 3:** Age-adjusted logistic regression analysis of sex differences in cardiometabolic risk factors Adjusted for age group (<50, 50-65, ≥65 years). Reference categories: male and <50 years. AOR: adjusted odds ratio from logistic regression, CI: confidence interval, BMI: body mass index, WC: waist circumference, WHR: waist-hip ratio, RBG: random blood glucose, HbA1c: glycated hemoglobin, BP: blood pressure

Outcome	Predictor	AOR (Exp B)	95% CI for AOR	p-value
BMI (kg/m²) (overweight/obese vs. normal)	Male (ref)	1.00	-	-
	Female	0.65	0.29-1.49	0.313
	Age <50 years (ref)	1.00	-	-
	Age 50-65 years	0.35	0.13-0.92	0.033
	Age >65 years	0.51	0.21-1.21	0.127
WC (cm) (high vs. normal)	Male (ref)	1.00	-	-
	Female	0.42	0.15-1.17	0.097
	Age <50 years (ref)	1.00	-	-
	Age 50-65	0.89	0.28-2.82	0.842
	Age >65	0.48	0.15-1.55	0.217
WHR (high/moderate risk vs. low risk)	Male (ref)	1.00	-	-
	Female	0.27	0.05-1.58	0.147
	Age <50 years (ref)	1.00	-	-
	Age 50-65	0.998	0.15-6.63	0.998
	Age >65	0.22	0.02-2.34	0.210
RBG (mmol/L) (high vs. normal)	Male (ref)	1.00	-	-
	Female	0.70	0.31-1.57	0.389
	Age <50 years (ref)	1.00	-	-
	Age 50-65	1.38	0.58-3.27	0.462
	Age >65	1.60	0.69-3.69	0.273
HbA1c (%) (high vs. normal)	Male (ref)	1.00	-	-
	Female	1.58	0.69-3.65	0.282
	Age <50 years (ref)	1.00	-	-
	Age 50-65	1.03	0.46-2.29	0.939
	Age >65	1.08	0.50-2.33	0.846
BP (mmHg) (uncontrolled/high vs. normal)	Male (ref)	1.00	-	-
	Female	1.77	0.75-3.65	0.211
	Age <50 years (ref)	1.00	-	-
	Age 50-65	1.27	0.54-2.99	0.589
	Age >65	0.78	0.34-1.78	0.557

## Discussion

This study aimed to determine sex differences in cardiometabolic risks among adults with T2DM attending a regional referral health facility in Kenya. The findings reveal notable disparities between males and females in the prevalence of key cardiometabolic risk factors, possibly related to genetic variations [17]. Significant sex differences were also observed across several sociodemographic characteristics. Most participants were female, and the mean age was 56.34 years. Significantly more males than females were of advanced age (>65 years) despite the participants being predominantly female. Past evidence suggests that women develop T2DM later in life due to postmenopausal changes. In contrast, men are diagnosed earlier, possibly linked to central obesity and lifestyle risks such as tobacco and alcohol use [18,19]. However, the findings in our study may be linked to delayed health-seeking behavior and accumulation of risks with age among male participants. After adjusting for age in logistic regression, the associations between sex and most cardiometabolic outcomes were attenuated, suggesting age as a partial confounder, particularly for central adiposity variables. Explicitly, sex was not independently associated with high BMI, WC, WHR, RBG, HbA1c, or BP.

Male participants were significantly more likely to be employed and to hold a higher employment status than female participants, suggesting a higher economic status. This may limit access to healthcare, the ability to afford medications, and engagement in self-management and follow-up. These findings are consistent with a Kenyan survey in which women with diabetes had higher unemployment rates and lower educational attainment, highlighting sex-related socioeconomic disparities that correlate with diabetes prevalence and self-care [11].

Marital status, which may be linked to the availability of support, also varied significantly by sex. Females were more frequently widowed, divorced, or single, while men were more often married. Support from significant others has been associated with improved self-management behavior in populations with diabetes and CVD, while social isolation or reduced support may negatively affect management outcomes [20]. Similarly, a study in rural China found that divorced or widowed status was positively associated with essential hypertension among people with diabetes, citing reduced support for self-management activities [21].

Contrastingly, religion did not differ significantly by sex, likely because most participants were Christian. Similarly, although there was a trend towards lower educational attainment among females, the difference was not statistically significant. This aligns with findings from a study in rural Kenya in which education and marital status were not associated with self-management practices among adults with T2DM [22]. However, other evidence suggests that higher educational attainment is associated with a lower diabetes burden, both in prevalence and management outcomes, likely due to better knowledge of prevention and care strategies [9,23]. The relationship between sex and sociodemographic factors highlights the importance of considering these variables when designing diabetes management strategies.

Our study found that at the time of recruitment, the majority of participants had been diagnosed with hypertension, a common risk factor for CVD. Similarly, a recent systematic review in Ethiopia reported a high prevalence of hypertension among patients with T2DM [24]. This co-occurrence of hypertension and diabetes may be linked to shared pathophysiology and risk factors such as aging and obesity. Moreover, most of those already diagnosed with hypertension were female, as observed in our study, possibly due to sex-specific differences in hypertension control, the higher age of female participants, and the predominantly female study population [25]. Previous studies in Kenya suggest that women over 30 years may have an increased risk of hypertension compared to men of similar age, which may explain the high hypertension burden in a predominantly female population in this study [18].

Although not statistically significant, more females than males had elevated BP readings in the current study. The higher prevalence of hypertension and elevated BP in female participants may relate to their older age and the shared pathophysiology and risk factors between diabetes and hypertension, as noted in another systematic review in Ethiopia [26]. Conversely, a prospective cohort study in the Netherlands found that men with T2DM were at greater risk of developing multiple microvascular complications, including hypertension. At the same time, women were more prone to macrovascular complications [27]. These contrasting findings may be attributable to differences in diabetes duration across study populations, as longer diabetes duration and older age are associated with increased risk of complications [28].

Additionally, BP did not differ by sex after adjustment for age, suggesting a more uniform risk across sexes in this cohort. These results, after adjusting for age, align with those from a prior study in a similar but larger cohort, in which no significant sex differences in BP were found after age adjustment [29]. This indicates that the outcome may be influenced more by factors such as treatment adherence and other clinical variables than by sex alone.

Similarly, when assessing for CVD risks using BMI and WHR classification, females exhibited a significantly higher prevalence of CVD risk factors than males. Females were more likely to be obese and to have a high WHR compared to males, likely due to differences in body fat distribution and hormonal effects, particularly postmenopausal changes, as highlighted in previous narrative reviews [7,8]. High WC and WHR indicate abdominal or central adiposity, which is linked to substantial cardiometabolic risk [30]. These findings align with other research documenting sex differences in CVD risk, with women at higher risk than men [4,5]. Conversely, previous evidence suggests that men are more prone to accumulate abdominal fat.

In contrast, women tend to store more fat around the gluteal region, which typically results in higher WC in men [29]. Estrogen influences fat distribution by regulating lipolysis and lipogenesis, which may explain sex-related disparities in fat distribution. These studies also agree that CVD prevalence is higher in males than in females earlier in life. Still, this trend reverses with age, likely due to the loss of estrogen’s protective effects after menopause [19].

An age-adjusted analysis of central adiposity measures indicates that age is a partial confounder. There was no statistically significant sex difference in WC after adjustment for age. However, the association was borderline, with female participants having lower odds of high WC than male participants. Despite the unadjusted χ² test indicating a strong sex difference in WHR, the age-adjusted logistic regression yielded a non-significant association, likely due to small cell counts within the high-risk WHR categories. BMI, however, was an exception in which age showed a significant independent effect. Participants aged 50-65 years had significantly lower odds of being overweight/obese compared with those aged <50 years, probably linked to age-related differences in body composition and disease duration, as well as lifestyle factors.

Additionally, younger adults may present with higher adiposity earlier in the course of diabetes. In comparison, older adults may experience weight loss from other chronic diseases, such as sarcopenia, or the effects of medication [31]. Furthermore, older adults with T2DM are likely to have a decline in muscle mass and an increase in fat mass with age, putting them at a higher risk for cardiometabolic risks despite similar or lower BMI measurements than the younger adults [32].

Our findings indicate a trend toward higher RBG and HbA1c levels in females than in males, although the differences were not statistically significant. This may be correlated with the higher obesity indices observed among female participants. Suboptimal glycemic control associated with overweight or obesity is often linked to insulin resistance [7]. Additionally, comorbidities such as hypertension, as observed in our study, can adversely affect glycemic control in T2DM. This explanation aligns with a study in Morocco that found that elevated BMI was more common among females and was significantly associated with poor glycemic control. In contrast, reductions in obesity were associated with improved glycemic control in patients with T2DM [33].

On the other hand, age-adjusted logistic regression analysis revealed that sex was not independently associated with poor glycemic control. Female participants had lower odds of elevated RBG and HbA1c compared with males; however, this difference was not statistically significant. These findings align with evidence from prior studies in which sex differences in glycemic control in T2DM remained modest or negligible after adjustment for confounders, suggesting differences in treatment response, context, or other unaccounted-for factors [34].

Diabetes presents significantly higher relative cardiometabolic risk in females than in males, likely due to biological factors. The predominantly female study population may also reflect higher health-seeking behavior and the tendency for diabetes to develop later in life. Furthermore, obesity and abdominal fat distribution are positively associated with CVD, and this pattern is commonly observed in females with diabetes [6]. The age factor is also important, as cardiovascular risk increases with age and the protective effect of estrogen is lost after menopause [19]. In fact, the age-adjusted logistic regression results underscore the importance of adjusting for age when assessing sex differences in cardiometabolic risks in DM. Multivariate analysis demonstrates that age and sample distribution partially explain the differences highlighted in the unadjusted χ² tests. However, ethnic, racial, and other social determinants of health across populations may explain disparities in trends in cardiometabolic risk [9].

Strengths and limitations of the study

This study highlights sex disparities in cardiometabolic risk profiles among adults with T2DM, providing valuable insights that may inform future management strategies. The inclusion of both clinical and anthropometric indices strengthens the assessment of cardiometabolic risk. Additionally, the use of standard procedures for data collection and measurements enhances the validity and reliability of the results.

However, the study has several limitations. Significant cardiometabolic risks such as dyslipidemia, smoking, and alcohol intake were not evaluated. Additionally, the reliance on RBG measurements to evaluate glycemic control is a limitation, as participants attended the outpatient clinic at different times, and researchers could not control for at-home dietary intake or glycemic management. HbA1c measurement partially addressed this limitation. Using clinic-based participants may introduce selection bias, as these individuals may be more aware of cardiometabolic risks than the general population. Despite maximal recruitment of eligible participants, the non-probability sampling method may have introduced recruitment bias. The predominantly female population may also influence the patterns of cardiometabolic risks. Finally, the cross-sectional design limits causal interpretations of the findings. Longitudinal studies are recommended to establish causal pathways for cardiometabolic risks and sex disparities.

## Conclusions

The sex disparities highlight the need for sex-responsive strategies in clinical practice, especially for managing T2DM and its complications. Females showed higher cardiometabolic risks, including BMI, WC, and WHR, compared with males. However, the logistic regression analysis highlights the importance of adjusting for age when assessing sex differences in cardiometabolic risk. Sex was not independently associated with BMI, WC, WHR, poor glycemic control (RBG and HbA1c), or high BP. However, older adults (50-65 years) had lower odds of high BMI than younger adults (<50 years), likely attributable to age-related changes in body composition and disease trajectories or durations. Significant sex differences in age, marital status, and employment status suggest that social determinants need to be considered in controlling cardiometabolic risks in diabetes. Managing these risks is crucial for diabetes control and prevention, as they are closely linked to glycemic control.

Targeted interventions that are age and sex responsive should address hypertension, BMI, WHR, and WC, as well as the socioeconomic barriers that may limit optimal management. This approach may inform broader national-level strategies to achieve universal health coverage in diabetes care, pending multi-site validation. Future studies should evaluate all cardiometabolic risk factors, including those not highlighted in this study.
